# Optimal Design of an Ultrasound Transducer for Efficient Acousto-Optic Modulation of Terahertz Radiation

**DOI:** 10.3390/ma15031203

**Published:** 2022-02-05

**Authors:** Pavel Alekseevich Nikitin, Vasily Valerievich Gerasimov

**Affiliations:** 1 Scientific and Technological Centre of Unique Instrumentation RAS, 117342 Moscow, Russia; 2 Moscow Power Engineering Institute, Krasnokazarmennaya Str. 14, 111250 Moscow, Russia; 3 Department of Physics, Novosibirsk State University, 630090 Novosibirsk, Russia; v.v.gerasimov3@gmail.com; 4 Budker Institute of Nuclear Physics SB RAS, 630090 Novosibirsk, Russia

**Keywords:** acousto-optic interaction, terahertz radiation, diffraction, liquefied inert gas

## Abstract

Acousto-optic (AO) interaction in the terahertz range was investigated with the use of monolithic ultrasound transducers of various widths. Sulfur hexafluoride (SF${}_{6}$) liquefied at a temperature of about 23 °C and a pressure of 25 bar was used as a medium for AO interaction. The angular and frequency bandwidths of effective AO interaction, as well as the diffraction efficiency per 1 W of the driving electric power, were determined. For the first time, a correct comparison of the AO diffraction efficiency in SF${}_{6}$ with the use of ultrasound transducers with different widths was carried out. In the experiments performed, the highest energy efficiency of the AO modulator was achieved with a transducer with a width of 12 mm.

## 1. Introduction

**Figure 1:**
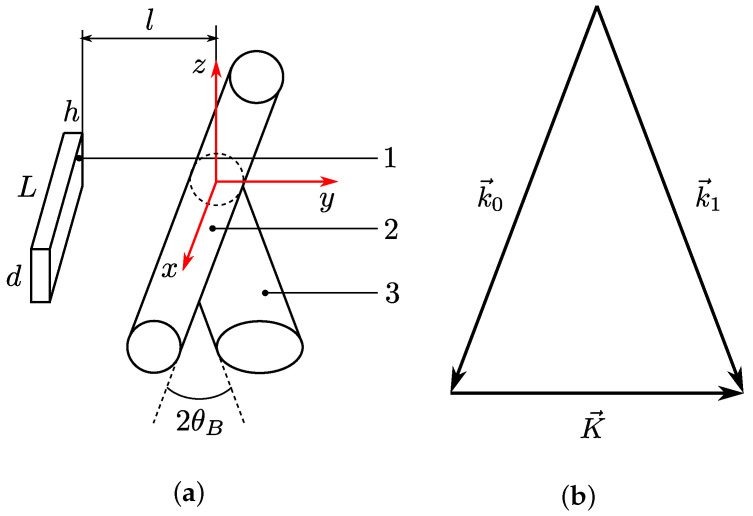
(**a**) Schematic of AO Bragg diffraction: 1—sound transducer; 2—transmitted radiation; 3—diffracted radiation; (**b**) wave vector diagram of AO interaction.

Acousto-optic (AO) interaction is an effective tool for modulation of laser beam intensity with a time response of about 1 μs [1,2]. The main concept is to use an acoustic wave to form a phase diffraction grating in a medium. Due to the photo-elastic effect, the refractive index variation is proportional to the amplitude of the acoustic wave. Therefore, by coding the electrical signal applied to the ultrasound transducer, one can modulate the intensity of the diffracted radiation. The best medium for AO interaction in the terahertz (THz) range is liquefied sulfur hexafluoride (SF${}_{6}$) [3,4]. In AO modulators, the Bragg regime was used and there were only two diffraction orders with wave vectors ${\vec{k}}_{0}$ and ${\vec{k}}_{1}$ (see Figure 1). The direction of ${\vec{k}}_{1}$ is determined by the relation ${\vec{k}}_{1}={\vec{k}}_{0}+\vec{K}$, where $\vec{K}$ is the wave vector of the sound wave.

The deflection angle between the transmitted and diffracted radiation beams equals the doubled Bragg angle $\theta_{B}$, which depends on the sound frequency *F*, radiation wavelength λ, and sound velocity *V* [5]:

(1) $$\sin\theta_{B}=\frac{K}{2k}=\frac{\lambda F}{2V}.$$

The diffracted radiation beam has to be deflected with an angle much larger than the divergence angle of this beam, which is about 1 degree for a beam diameter of 10 mm and a wavelength of 130 μm. The sound velocity in liquefied SF${}_{6}$ is about V≈300 m/s. Therefore, at the sound frequency F=300 kHz, the deflection angle is about 8 degrees, which is sufficient for its spatial separation from the transmitted radiation beam. The use of higher frequencies is not advisable because of the high attenuation of ultrasound, which is proportional to the square of the frequency. The resonant frequency $F_{\mathrm{res}}=V_{\mathrm{PZT}}/2h$ of sound transducers is proportional to the sound velocity $V_{\mathrm{PZT}}$ in piezoceramics and is inversely proportional to its thickness *h*. The typical sound transducer thickness is about h≈6 mm for $F_{0}=300$ kHz [3,4]. The sound transducer width *d* should not be much greater than the diameter of the radiation beam, which is about 1 cm for the Novosibirsk free-electron laser (FEL) [6]. Hence, the sound transducer width *d* is close to its thickness *h*. As known, with d/h≈1, complex types of mechanical vibrations arise in the transducer [7,8]. As a result, the wavefront of the generated sound wave can no longer be considered as flat, which negatively affects the efficiency of the AO interaction. In addition, with a transducer the width of which exceeds the diameter of the radiation beam, part of the sound beam does not interact with the radiation beam, and the efficiency of the AO diffraction becomes lower. Therefore, there is an optimal width of the transducer at which the diffraction efficiency reaches its maximum.

According to the simplest model, the real sound beam is replaced by a homogeneous sound column with a plane wave front [9]. In this case, due to a decrease in the acoustic power density, the AO diffraction efficiency is inversely proportional to the width of the ultrasound transducer. Despite the fact that this model seems to be crude, it describes well the results of most known works on acousto-optics [10,11,12]. This is because of the common use of high frequency ultrasound of about 100 MHz and above. As a result, the thickness of the ultrasound transducer is very small and is about $h=V_{\mathrm{PZT}}/2F\approx 100\;\mu$m, which is 2 orders of magnitude less than its width (about several millimeters) and length (about 1 cm). Therefore, such transducers behave like a piston. Since up to now there was no need to use thick ultrasound transducers, the simplest model was used and it was not necessary to take into account the influence of the transducer thickness on the characteristics of AO devices.

As mentioned above, for the effective AO modulation of THz radiation, it is necessary to use liquefied SF${}_{6}$ as the medium for the AO interaction. As a result, the thickness of the transducer is comparable to its width. As far as we know, there are only a few works in which the AO diffraction of THz radiation in liquefied SF${}_{6}$ [3,4,13] was investigated. The results obtained in these works cannot be compared correctly because of the lack of data about the active electrical power consumed by the sound transducer. The purpose of this work is to find the optimal width of the ultrasound transducer to increase the energy efficiency of the AO modulator of THz radiation based on liquefied SF${}_{6}$.

## 2. Materials and Methods

### 2.1. Theoretical Background

In the Bragg regime of AO diffraction, only two diffraction maxima are observed: Zero and the first, with the intensities $I_{0}$ and $I_{1}$, respectively. As a measure of the efficiency of AO diffraction, the ratio $I_{1}/I_{0}$ is usually used. The power consumption required to achieve a given level of modulation depth is also important. Therefore, the diffraction efficiency ξ is normalized to the active electrical power $P_{\mathrm{el}}$ [14,15]:

(2) ξ=I1I0,ξnorm=ξPel,Pel=12U2Re(Z)|Z|2,

where $\xi_{\mathrm{norm}}$ is the diffraction efficiency per 1 W of the electrical power; *U* is the voltage amplitude on the sound transducer; Re(Z) and |Z| are the real part and absolute value, respectively, of the frequency dependent impedance of the sound transducer.

To estimate the efficiency of AO diffraction, one can use the relation obtained within the simplest model of AO interaction [14] (which assumes the diffraction of a plane electromagnetic wave by an infinite sound column), introducing in it the additional exponential term considering the ultrasonic attenuation:

(3) $$\xi_{1D}=\frac{\pi^{2}}{2\lambda^{2}}\frac{M_{2}P_{a}}{d}L\exp\left(-\alpha_{s}l\right),$$

where $M_{2}$ is the AO figure of merit; $P_{a}$ is the acoustic power, which is usually considered as equal to the RF driving power $P_{\mathrm{el}}$; *d* and *L* are the width and length of the sound transducer; $\alpha_{s}$ is the sound power attenuation coefficient; *l* is the distance from the sound transducer at which the THz beam travels (in our experiment l=5 cm). The dependence of physical properties of liquefied SF${}_{6}$ on the temperature and pressure are summarized in work [4].

As one can see from Equations (2) and (3), the diffraction efficiency ξ is proportional to the RF driving power $P_{\mathrm{el}}$, whereas the normalized diffraction efficiency $\xi_{\mathrm{norm}}$ does not depend on $P_{\mathrm{el}}$:

(4) $$\xi =k_{P}P_{\mathrm{el}},$$

where the factor $k_{P}$ can be determined from experimental data by the least square method (LSM).

The diffraction efficiency ξ in a resonant manner depends on the angle of incidence θ of radiation on the AO cell, as well as on the ultrasound frequency *F*. For the interaction between plane electromagnetic and acoustic waves, this dependence has the form of sinc${}^{2}\left(x\right)$, whose argument is proportional to the deviation of θ or *F* from Bragg condition (1) [16]. The two-sided interaction bandwidths (−3 dB criterion for the normalized diffraction efficiency $\xi_{\mathrm{norm}}$) can be calculated with the use of the known relations [17]:

(5) $$\begin{array}{ccc} \; & \Delta \theta =\frac{0.9nV}{FL_{\mathrm{eff}}}, & \Delta F=\frac{1.8nV^{2}}{\lambda FL_{\mathrm{eff}}}, \end{array}$$

where $L_{\mathrm{eff}}$ is the effective length of the AO interaction region, which is usually equal to or slightly shorter than the length of the sound transducer *L*. The relation for the angular bandwidth differs from the original one in [17] by the value *n*, since in [17] the angle is calculated for the interaction medium in the AO cell, while in this work the angle is calculated outside the cell.

**Figure 2:**
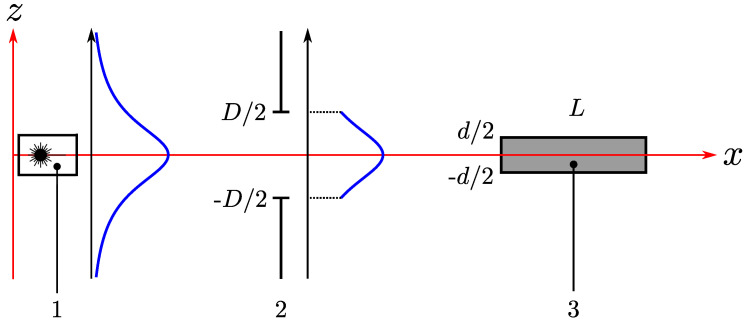
Schematic of acousto-optic Bragg diffraction: 1—THz laser; 2—optical window of AO cell; 3—region of AO interaction.

Equation (3) was derived for the diffraction of plane electromagnetic wave. However, it is necessary to take into account the size *d* of the AO interaction region in *z*-direction and the size *D* of the optical window of the AO cell in *z*-direction (see Figure 2). If the optical window can be considered as infinitely wide in the direction of the *y* axis, the mathematical solution does not depend on *y* and we can use a one-dimensional distribution of the radiation beam intensity instead of a two-dimensional one.

The intensity distribution of the THz beam is well fitted by the Gaussian function with amplitude *A* and width $\omega_{z}$ [6]. In the one-dimensional case, it can be written as follows:

(6) $$I_{\mathrm{THz}}\left(z\right)=A\exp\left(-\frac{z^{2}}{\omega_{z}^{2}}\right).$$

It can be shown that the integral intensities of the transmitted and diffracted beams can be calculated by the following relations:

(7) $$I_{0}=\exp\left(-\alpha L\right)\int_{-D/2}^{D/2}I_{\mathrm{THz}}\left(z\right)dz;$$

(8) $$I_{1}=\left\{\begin{array}{cc} \xi_{1D}\exp\left(-\alpha L\right)\int_{-d/2}^{d/2}I_{\mathrm{THz}}\left(z\right)dz & \mathrm{for}\;d\leq D;\\ \xi_{1D}\exp\left(-\alpha L\right)\int_{-D/2}^{D/2}I_{\mathrm{THz}}\left(z\right)dz & \mathrm{for}\;d>D. \end{array}\right.$$

The derivation of relations (7) and (8) requires some explanation. The integral intensity $I_{0}$ of the transmitted radiation is limited only by the optical window size *D*. Therefore, the integration limits are −D/2 and *D*/2. The exponential term before the integral corresponds to the light absorption in the medium. The integral intensity $I_{1}$ of the diffracted radiation is proportional to the integral intensity of the THz beam in the region of the AO interaction. So, the limits in the integral are determined by the smallest of the optical window size *D* and the sound transducer width *d*.

Let us give an example of calculations for an experiment that uses a THz laser with a wide beam of radiation, such as the Novosibirsk free electron laser ($\omega_{z}=1.44$ cm for λ=130μm and 8–11 stations) [6]. The minimum size of the optical window is limited by the enhancement of the effect of radiation diffraction at the edges of the optical window. Therefore, we assume D=1 cm, as in the experiment in work [4]. As the variation of $I_{\mathrm{THz}}\left(z\right)$ for |z|<0.5 cm is less than 10%, this function can be treated as constant. As a result, the integrals in (7) and (8) are proportional to the integration interval width. The diffraction efficiency can now be determined with the use of relation (2) in the following way:

(9) $$\xi =\left\{\begin{array}{cc} \xi_{1D}\frac{d}{D} & \mathrm{for}\;d\leq D;\\ \xi_{1D} & \mathrm{for}\;d>D. \end{array}\right.$$

**Figure 3:**
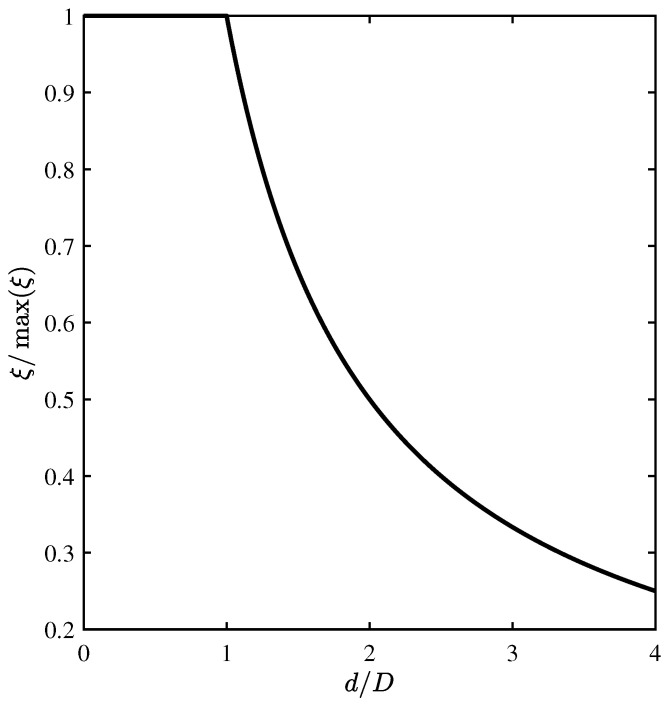
Theoretical dependence of diffraction efficiency on width of sound transducer.

The dependence ξ(d) is a piecewise function, which is shown in Figure 3.

It can be seen from (9) that when the radiation beams wider than the sound transducer are used, the diffraction efficiency decreases by a factor of d/D. It is related to the fact that part of the radiation passes away from the sound beam and does not interact with it. This factor is limiting and inhibits infinite increase in the efficiency of AO diffraction due to decrease in the width *d* of the sound transducer, as follows from (3).

### 2.2. Experimental Technique

**Figure 4:**
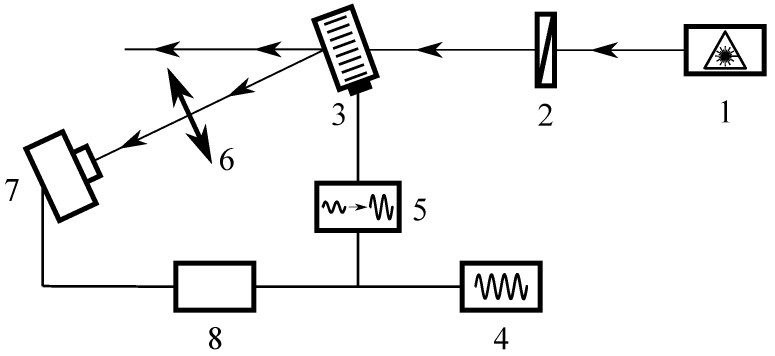
Schematic diagram of experimental setup: 1—FEL; 2—wire-grid polarizer; 3—AO cell; 4—signal generator; 5—electrical amplifier; 6—lens; 7—Golay cell; 8—lock-in amplifier.

Monochromatic coherent THz radiation of the Novosibirsk free electron laser 1 (FEL) with the wavelength λ=130μm (see Figure 4) was used in our experiments. Since an AO modulator based on a liquid medium is insensitive to radiation polarization, the intensity of the THz beam was set by wire polarizer 2. The radiation beam was incident on the center of the input optical window of AO cell 3 (D=10 mm) at the distance l=5 cm from the sound transducer. A detailed description of the AO cell is given in [13]. For observation of the diffracted radiation, the AO cell was turned through the Bragg angle, and a signal (modulated at a frequency of 10 Hz) from generator of electrical signals 4 was applied to the ultrasound transducer via amplifier 5. At a distance of about 30 cm after the AO cell, lens 6 was located, focusing the radiation into the receiver, Golay cell 7. The signal from the receiver was isolated from the background noise by means of lock-in amplifier 8.

The cycle of experiments can be broken into the following stages:

1. At the first stage, the setup was adjusted via change in the sound frequency *F* and the angle of incidence of THz radiation on the AO cell. The aim was to achieve the maximum intensity of the diffracted radiation.
2. Next, the dependence $I_{1}\left(U\right)$ of the intensity of the diffracted radiation on the amplitude of the electric signal was measured. In theory, this dependence is quadratic.
3. Further measurements were carried out at the maximum level of the voltage amplitude *U*, at which the $I_{1}\left(U\right)$ dependence is still quadratic.
4. The dependence $I_{1}\left(\theta \right)$ of the intensity of the diffracted radiation on the angle of incidence of the radiation was measured. The angle of incidence was changed via rotation of the AO cell.
5. Finally, the dependence $I_{1}\left(F\right)$ of the diffracted radiation intensity on the ultrasound frequency was measured at variation of the frequency of the electrical signal applied to the sound transducer.
6. Since the electrical impedance *Z* of the ultrasound transducer depends on the frequency, the amplitude *U* of the electrical signal was changed together with the frequency. Therefore, the dependencies Z(F) and U(F) were measured too.
7. The diffraction efficiency was normalized to 1 W of the electrical power for determination of the energy efficiency of the AO modulator.

The measurement error for the diffraction efficiency ξ was mainly caused by the FEL radiation intensity instability of about 10%. The sound frequency *F* was set with an accuracy of 0.001 kHz, while the angle θ with an accuracy of 0.5′. The temperature *t* and pressure *p* were measured with an accuracy of 0.5 °C and 0.5 bar, respectively. To avoid cavitation near the surface of the ultrasound transducer, we worked in a mode where the electrical power consumption was low (about 1 W) and the efficiency of AO diffraction was proportional to the square of the voltage on the transducer.

## 3. Results and Discussion

**Figure 5:**
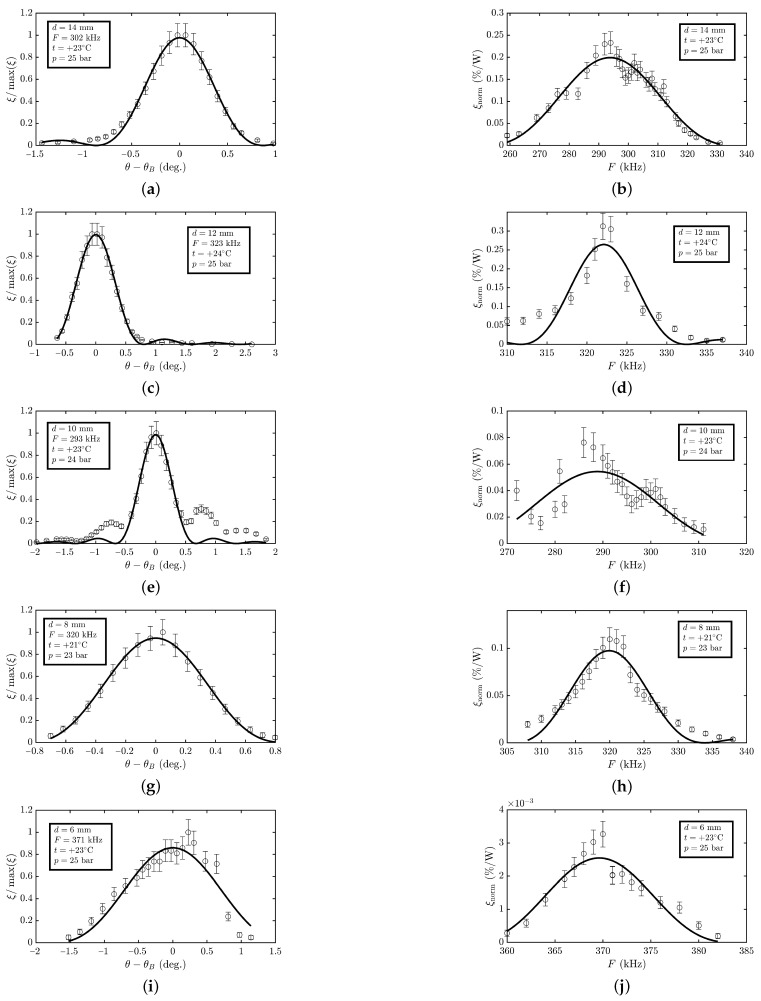
Experimental results: (**a**,**c**,**e**,**g**,**i**) AO diffraction efficiency ξ/max(ξ) vs. difference between angle θ of incidence of THz radiation on AO cell and Bragg angle $\theta_{B}$; (**b**,**d**,**f**,**h**,**j**) frequency dependence of AO diffraction efficiency per 1 W of input electric power.

**Figure 6:**
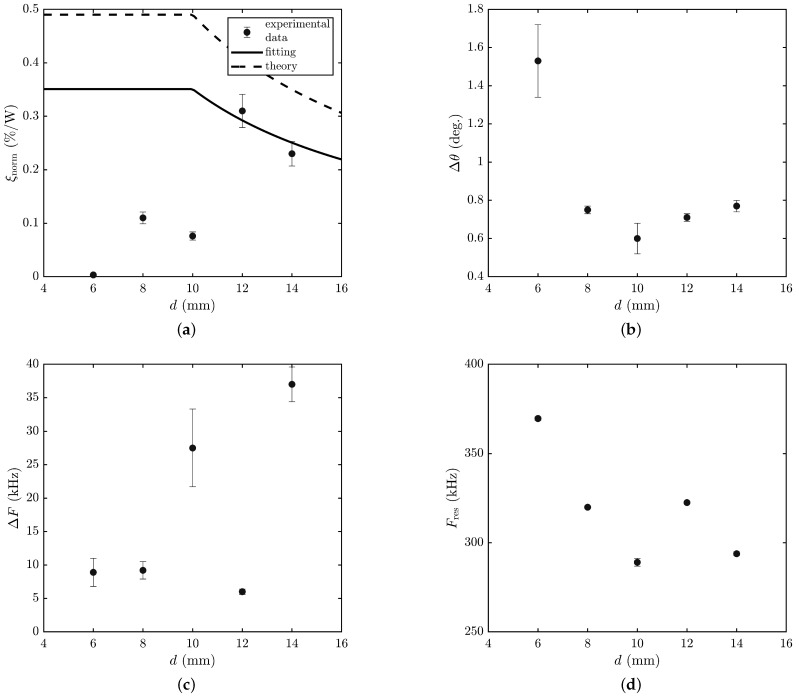
Dependence of parameters of AO modulator on width *d* of ultrasound transducer: (**a**) AO diffraction efficiency per 1 W of input electric power; (**b**) angular bandwidth; (**c**) frequency bandwidth; (**d**) operating frequency.

The measured dependencies were approximated by the least squares method in accordance with the theoretical model and are shown in Figure 5. The error bars correspond to the experimental errors. As expected, they are of resonant nature, which made it possible to determine the working bandwidth of the angles of incidence of radiation and the working bandwidth of the ultrasound frequency. Table 1 shows the values of the parameters of the AO modulator based on liquefied SF${}_{6}$ gas, obtained with various ultrasound transducers with a width *d* of 6 to 14 mm. The results for *d* = 14 mm was taken from our previous work [13]. For clarity, the results are presented in the form of graphs in Figure 6.

The measured dependencies (see Figure 5a,c,e,g,i) of the diffraction efficiency $\xi_{\mathrm{norm}}$ on the angle θ of incidence of radiation on the AO cell are fairly well described by a theoretical dependence of the form sinc${}^{2}\left(x\right)$. At the same time, some of them have pronounced side lobes, while others are of asymmetrical shape. This can be explained by the fact that the sound beam features a directional diagram, in which there are components propagating at an angle to the normal of the ultrasound transducer. In addition, the asymmetry of the frequency dependencies (see Figure 5b,d,f,h,j) of the AO diffraction efficiency indicates a complex structure of the acoustic modes of the transducer due to the fact that the width *d* of the ultrasound transducer is comparable to its thickness (6 mm). This is confirmed, among other things, by the dependence of the resonant frequency $F_{\mathrm{res}}$ of the ultrasound transducer on its width *d* (see Figure 6d).

The angular Δθ and frequency ΔF interaction bandwidths were expected to not depend on the width of the ultrasound transducer and be determined only by the effective length of AO interaction $L_{\mathrm{eff}}$ (see Equation (5)). However, as can be seen from Figure 6b,c, this is not the case: With a decrease in the width *d* of the ultrasound transducer, the working bandwidth Δθ of the incidence angles of THz radiation increases, while the working frequency bandwidth ΔF of ultrasound, in contrast, decreases. These facts once again confirm that an ultrasound transducer with a width almost equal to its thickness behaves in a complex manner, and its behavior can no longer be described by the approximation of a piston-type transducer [18].

In accordance with Figure 3, the highest efficiency of AO diffraction would be achieved at d<D, i.e., at d<10 mm, and at d>D; the AO diffraction efficiency would decrease according to the law ξ∝1/d. As can be seen from Figure 6a, with the ultrasound transducer width d=12 mm and 14 mm, the model agrees well with the experimental results. However, for d=10 mm and less, the experimental data differ significantly from the theoretical model. This can be explained by the influence of two factors. Firstly, the model assumes that the radiation is diffracted by a sound beam with a plane wavefront. However, at the frequency F=300 kHz, the wavelength of sound in liquefied SF${}_{6}$ is about 1 mm due to the low speed of sound of about 300 m/s. Therefore, at d=6 mm, only 6 wavelengths fit on the ultrasound transducer, which leads to a strong diffraction divergence of the sound beam. Secondly, at d=6 mm, the ultrasound transducer has a square cross-section, which leads to complex deformations when an alternating voltage is applied to the electrodes. As a result, the wavefront is also strongly distorted, which leads to a decrease in the AO diffraction efficiency.

## 4. Conclusions

Many parameters of AO devices designed for radiation in the ultraviolet, visible, and infrared spectral ranges, such as the angular Δθ and frequency ΔF bandwidths of effective interaction, as well as the operating frequency $F_{\mathrm{res}}$, do not depend on the width *d* of the ultrasound transducer. However, our study has shown that this is not the case for AO modulators of THz radiation based on liquefied SF${}_{6}$. It has been found found that with a decrease in the width *d* of the ultrasound transducer from 14 to 6 mm, the frequency bandwidth ΔF decreases. The angular bandwidth Δθ is approximately constant in the interval of *d* from 8 to 14 mm and increases by 2 times at d=6 mm. At the same time, as the transducer width *d* decreases from 14 mm to 6 mm, the AO diffraction efficiency decreases by about two orders of magnitude. Such unusual characteristics, in our opinion, can be explained by the fact that the ultrasound transducer applied was “thick”. This led to the complex deformations of the transducer and, as a consequence, to phase inhomogeneities of the ultrasonic field. Therefore, when fabricating THz radiation modulators based on liquefied SF${}_{6}$ gas with an operating frequency of about 300 kHz, it is advisable to use an ultrasound transducer with a width *d* of about 12 mm. In this case, its characteristics are more predictable, and the diffraction efficiency $\xi_{\mathrm{norm}}$ reaches its maximum value.

## Figures and Tables

**Table 1:** Properties of AO modulator of THz radiation revealed in the experiment.

*d* (mm)	ξ_norm_ (%/W)	Δθ (deg)	ΔF (kHz)	$F_{\mathrm{res}}$ (kHz)
14	0.23±0.02	0.77±0.03	37.0±2.6	293.8±1.1
12	0.31±0.03	0.71±0.02	6.0±0.4	322.5±0.5
10	0.076±0.007	0.60±0.08	27.5±5.8	289.0±2.2
8	0.11±0.01	0.75±0.02	9.2±1.3	319.9±0.5
6	$\left(3.3\pm 0.3\right)\times 10^{-3}$	1.53±0.19	8.9±2.1	369.6±0.9

## Data Availability

The data presented in this study are available on request from the corresponding author. Because of the further research, the data are not publicly available.

## Abbreviations

The following abbreviations are used in this manuscript:

AO	acousto-optic
THz	terahertz
FEL	free-electron laser
